# Classification of Asthma Based on Nonlinear Analysis of Breathing Pattern

**DOI:** 10.1371/journal.pone.0147976

**Published:** 2016-01-29

**Authors:** Mohammad Reza Raoufy, Tara Ghafari, Reza Darooei, Milad Nazari, Seyed Alireza Mahdaviani, Ali Reza Eslaminejad, Mehdi Almasnia, Shahriar Gharibzadeh, Ali R. Mani, Sohrab Hajizadeh

**Affiliations:** 1 Department of Physiology, Faculty of Medical Sciences, Tarbiat Modares University, Tehran, Iran; 2 Department of Physiology, Medical School, Shahid Beheshti University of Medical Sciences, Tehran, Iran; 3 Department of Biomedical Engineering, Faculty of Electrical and Computer Engineering, Tarbiat Modares University, Tehran, Iran; 4 Faculty of Electrical Engineering, Sharif University of Technology, Tehran, Iran; 5 Pediatric Respiratory Diseases Research Center, National Research Institute of Tuberculosis and Lung Diseases (NRITLD), Shahid Beheshti University of Medical Sciences, Tehran, Iran; 6 Tracheal Diseases Research Center, National Research Institute of Tuberculosis and Lung Diseases (NRITLD), Shahid Beheshti University of Medical Sciences, Tehran, Iran; 7 Chronic Respiratory Diseases Research Center, National Research Institute of Tuberculosis and Lung Diseases (NRITLD), Shahid Beheshti University of Medical Sciences, Tehran, Iran; 8 Department of Bioelectric, Faculty of Biomedical Engineering, Amirkabir University of Technology, Tehran, Iran; 9 Division of Medicine, UCL, London, United Kingdom; University of Washington, UNITED STATES

## Abstract

Normal human breathing exhibits complex variability in both respiratory rhythm and volume. Analyzing such nonlinear fluctuations may provide clinically relevant information in patients with complex illnesses such as asthma. We compared the cycle-by-cycle fluctuations of inter-breath interval (IBI) and lung volume (LV) among healthy volunteers and patients with various types of asthma. Continuous respiratory datasets were collected from forty age-matched men including 10 healthy volunteers, 10 patients with controlled atopic asthma, 10 patients with uncontrolled atopic asthma, and 10 patients with uncontrolled non-atopic asthma during 60 min spontaneous breathing. Complexity of breathing pattern was quantified by calculating detrended fluctuation analysis, largest Lyapunov exponents, sample entropy, and cross-sample entropy. The IBI as well as LV fluctuations showed decreased long-range correlation, increased regularity and reduced sensitivity to initial conditions in patients with asthma, particularly in uncontrolled state. Our results also showed a strong synchronization between the IBI and LV in patients with uncontrolled asthma. Receiver operating characteristic (ROC) curve analysis showed that nonlinear analysis of breathing pattern has a diagnostic value in asthma and can be used in differentiating uncontrolled from controlled and non-atopic from atopic asthma. We suggest that complexity analysis of breathing dynamics may represent a novel physiologic marker to facilitate diagnosis and management of patients with asthma. However, future studies are needed to increase the validity of the study and to improve these novel methods for better patient management.

## Introduction

Human breathing dynamics reveal complex pattern of variations that is related to multiple feedback loops that interact with the internal and external stimuli to optimize the efficiency of gas exchange [1]. Understanding such nonlinear behavior may provide physiological insight to the respiratory system and may be used as a tool for clinical assessment of respiratory disorders [2]. Although nonlinear properties of the respiratory rhythm has extensively been studied in healthy individuals [2], the complexity of breathing dynamics in patients with lung diseases has been investigated in a limited number of studies.

Asthma is a complex disorder that involves multiple interactions between intrinsic and extrinsic factors [3, 4]. Clinically, the disease is divided into atopic and non-atopic asthma and, based on the level of response to therapy, is classified into controlled and uncontrolled asthma [4, 5]. Although several studies in the last decades have increased our insight into the mechanism of bronchial constriction/inflammation in asthma, a significant number of patients do not fully respond to available bronchodilator/anti-inflammatory treatments [4]. Rather, the pathophysiology of non-atopic asthma is poorly understood and may require a novel experimental approach [6]. It seems that nonlinear dynamics is useful for explaining the complexity of breathing pattern in asthma [3, 7, 8]. For instance, previous studies demonstrated that the irregularity of airflow pattern is decreased in asthmatic patients [7] and the loss of fractal-like correlations in day-to-day fluctuations of peak expiratory flows augments the risk of unstable airway function [3]. There is also evidence to show that respiratory variability analysis can distinguish patients with atopic from non-atopic asthma [8].

A valid description of the complex breathing dynamics in asthmatic patients requires continuous monitoring of the spontaneous breathing pattern using non-invasive tools. Moreover, fluctuations in the lung volume may significantly alter the airways response to broncho-active mediator [9]. Therefore, concurrent volume and rate variability analysis is crucial for assessing the respiratory dynamics in health and disease. Novel computational methods allow quantifying fractal-like structure of physiological rhythms as well as the level of synchronization between rate and volume [2, 10]. In the present study, we compared respiratory inter-breath interval (IBI) as well as lung volume (LV) fluctuations among healthy volunteers and asthmatic patients to differentiate various types of asthma.

## Material and Methods

### Data collection

Forty age-matched men including 10 healthy volunteers, 10 patients with controlled atopic asthma (CAA), 10 patients with uncontrolled atopic asthma (UAA), and 10 patients with uncontrolled non-atopic asthma (UNAA), ages 21 to 39 years, referred to the outpatient clinic of Masih Daneshvari Lung Hospital (Tehran, Iran) from June 2010 to February 2011, were enrolled in this study, as previously described [8, 11]. All participants signed informed written consent prior to data collection. The study was approved by the institutional review board and ethics committee at Tarbiat Modares University. Asthma was categorized as controlled and uncontrolled based on the National Asthma Education and Prevention Program (NAEPP) guidelines [5]. Atopic asthma was diagnosed based on the results of skin tests and clinical symptoms. Patients had no history of smoking, other respiratory diseases or a chronic medical problem (neurological impairment, cardiovascular disease, etc.), and were medication-free for at least 12 hours before recording.

**Fig 1:**
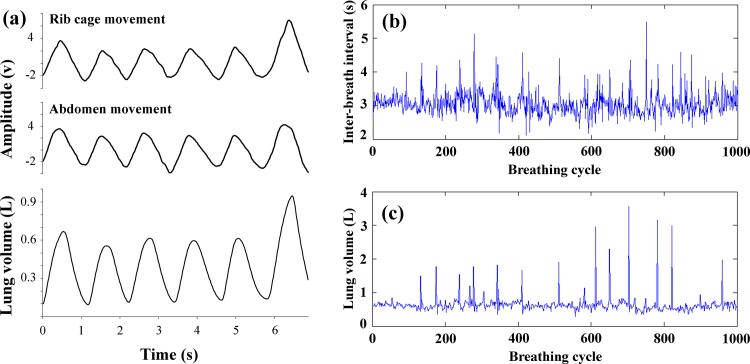
Breathing pattern in a representative subject. (a), An experimental tracing of abdominal and rib cage movement signals recorded continuously by pneumotrace bands (only a few seconds of tracing is presented for clarity). The plethysmography signals were calibrated to volume using an artificial neural network model. (b) and (c), Original (“raw”) inter-breath interval (b) and lung volume (c) time series during 60 min of resting breathing in a representative subject.

Subjects laid supine for about 70 min while continuous respiration signals were collected, using a respiratory inductive plethysmography. Two pneumotrace bands (AD-Instruments, Australia) were fastened at the level of umbilicus and fourth intercostal interspace, for monitoring the rib cage and abdomen movements. The plethysmography signals were calibrated to volume using an artificial neural network (ANN) system (Fig 1A), as previously described [11]. Briefly, for calibration purpose, abdominal and rib cage movement signals (as inputs of ANN) and respiratory volumes (as output of ANN) were simultaneously recorded during last about 7 minutes of each trial. Volume was measured with a spirometer (Pony Spirometer, Cosmed, Italy) which was connected to a digitizer device via an interface. The signals from the pneumotrace bands and spirometry were digitized at a 1 KHz sampling rate (Powerlab, ADInstruments, Australia). We used five minutes of data for developing model and the validation assessment was based on at least 40 following breaths at the same period. We have designed a standard feed-forward ANN (in MATLAB 7.4 environment, using its neural network toolbox), including eight input neurons, fifteen neurons in hidden layer, and one output neuron. The tansig and purelin functions were used for hidden layers and output layer respectively. We used the newff function to create the network object in training feed forward network and the Levenberg–Marquardt (trainlm) algorithm to train the back-propagation network. The ANN was trained 500 times (epochs). The validated ANN model was used for calibration of remaining plethysmography signals to volume.

We were observing the subjects during the recording in order to recognize the artifacts due to subjects’ movements, sneezes, coughs, etc. After omitting the artifacts, the IBI and LV time series were calculated for 60 min using commercially available software (Chart 5, ADInstrument, Australia) (Fig 1B and 1C). The peaks of each calibrated signal were detected and visually verified, and then the peak-to-peak intervals and the amplitude of peaks were considered as the IBI and LV series. In order to allow comparisons of data sets with different degrees of variability, all time-series were normalized to have a mean of zero and standard deviation = 1 as described [10].

### Time-series analysis

#### Detrended Fluctuation Analysis (DFA)

DFA permits the detection of long-range correlations within a time-series and is increasingly gaining attraction in the study of physiological signals [12]. A detailed description of the DFA algorithm appears elsewhere [12]. Briefly, the IBI and LV time-series after integration were divided into boxes of equal length, n. The integrated time-series were then detrended by subtracting the local trend in each box. This procedure was repeated for all different boxes. The variability is depicted on a log-log scale as a function of different sizes of boxes. A linear relationship between log(n) and log(variability) indicates the presence of fractal dynamics. The slope (α) of this line indicates the degree of long-range correlations. An α = 1 indicates perfect log-range correlation at different scales (Power law distribution with 1/f dynamics). A deviation from 1 in either direction represents a breakdown from 1/f fractal-like dynamics. An α = 0.5 corresponds to a random, uncorrelated process, i.e. white noise, whereas α = 1.5 corresponds to the integrated random walk, i.e. Brownian noise.

#### Sample Entropy (SampEn)

Sample entropy (SampEn) represents the degree of irregularity of a time-series. In other words, SampEn is the negative natural logarithm of the conditional probability that 2 sequences similar for m points remain similar at the next point with a tolerance r, where self-matches are not included [10,13]. For entropy analysis of IBI and LV time-series, different values of parameters (m, r, N) are used for calculations, where N is the length of the time-series, r is the tolerance for accepting matches, and m (embedding dimension) is the length of sequences to be compared [13]. In our analysis, we computed IBI and LV SampEn assigning the values of 2 for m and 0.2 for r, using MATLAB code available from the physionet (http://www.physionet.org).

#### IBI and LV cross-sample entropy

Cross-sample entropy (cross-SampEn) determines the degree of asynchrony of two distinct but interacting time series in a network [10, 13]. A higher degree of asynchrony indicates fewer sub-pattern matches, as quantified by larger cross-SampEn values. In contrast, lower values are indicative of stronger synchronization [10, 13]. We measured cross-SampEn after setting the values of m (2) and r (0.2) for quantifying asynchrony between IBI and LV time-series, using MATLAB code available from the physionet (http://www.physionet.org).

#### Largest Lyapunov Exponents (LLE)

Chaotic systems are considered to be sensitive to the initial conditions. LLE can assess the sensitivity to initial conditions and characterizes the divergence of nearby trajectories in the phase space. Briefly, consider two points in adjacent trajectories-states of the phase space, and assume the distance between them to be d(0). After time t, the average divergence can be written as d(t) = d(0) e ^LLE (iΔt)^, where LLE is the largest Lyapunov exponent of the system. For the LLE calculation, embedding dimension (m) and time delay (τ) values of each time series were estimated by using the false nearest neighbor [14] and average mutual information [15] methods, respectively. After determining the proper embedding dimension (m = 3.4 ± 1.5) and delay (τ = 1.3 ± 0.9), we computed LLE of the IBI and LV time-series, using the algorithm proposed by Rosenstein in MATLAB, which seems to be useful, particularly in small data sets [16]. Values higher than 0 reflect an unstable and unpredictable system, where nearby points will diverge to any arbitrary separation. Increased LLEs reflect increased sensitivity to initial conditions and characterize unpredictable variations, whereas low values indicate regularity [16].

### Classification method

We analyzed the performance of the combination of the complexity indices in the diagnosis of various types of asthma using weighted sparse representation based classification (WSRC) method which is a modified version of sparse representation classification (SRC) (S1 File). Briefly, the main idea of SRC is to represent new sample using the least number of training samples [17]. The problem of searching for sparse representation of coefficients vector x can be written by using equation y = Ax. In this equation, A is a matrix with *m* × *n* dimensions that includes *n* training samples (*m* < *n*), y is a new sample with unknown class label that requires being determined [17], and x is reconstructed by solving the convenient optimization problem:

$$\hat{\text{x}}=\text{arg}\;\underset{\text{x}}{\text{min}}{\left\|\text{x}\right\|}_{0}\;\text{subject to y}=\text{A}x$$

SRC has a drawback with classifying the data with the same direction distribution [18] and also needs sufficient training samples [19]. For eliminating these limitations, we used WSRC which remedies the drawback of SRC and is also more effective for small training sample sizes [20]. A geometric characterization of the samples defines as w = [*w*_1_, *w*_2_, …, *w*_*n*_]^*T*^, where w is the Minkowski distance among samples. The problem can be modified as:

$$\hat{\text{x}}=\text{arg}\;\underset{\text{x}}{\text{min}}\sum_{k=1}^{n}w_{k}\;{\left|x_{k}\right|}_{0}\;\text{subject to y}=\text{Ax}$$

In this study, leave-one-out cross-validation was performed for evaluating the classification performance of WSRC method. The function was trained *n* separate times (where *n* is the number of samples) on all the data except one sample in each iteration for which a prediction was made. The average error was calculated to evaluate the performance of WSRC [21].

Sequential Forward Selection (SFS) method is used to assess the overfitting. Firstly, an empty feature subset is considered; then, a feature which provides the best combination with the already selected features is added in from the rest of the features. This process is continued until all the features are selected [22] (More details in S1 File).

### Statistical analysis

We used GraphPad Prism V3.0 (GraphPad Software, San Diego, CA) for statistical analysis of data. A one-way ANOVA with a Bonferroni post-test or a Kruskal–Wallis non-parametric test with a Dunn’s post-test was used to compare the complexity indices of groups. Receiver Operating Characteristic (ROC) curves were used to evaluate the power of nonlinear methods for discriminating asthmatic patients (n = 30) from healthy volunteers (n = 10), uncontrolled (n = 20) from controlled (n = 10) and non-atopic (n = 10) from atopic (n = 20) asthmatic patients. P-values less than 0.05 were considered statistically significant.

## Results

### Study population and cycle-by-cycle variations in respiration

Forty men including healthy, CAA, UAA, and UNAA subjects were enrolled in the study. Each group had 10 subjects, and there were no significant differences in age (27.6±5.3, 30.8±9.8, 31.1±7.2, and 32.7±8.1, respectively; p = 0.526) and body mass index (BMI) (22.7±1.6, 22.2±2.1, 22.6±1.5, and 22.9±1.7, respectively; p = 0.845) among the groups. Table 1 demonstrates the average and the coefficient of variation (CV) of the IBI and LV series for all subjects. There were no significant differences in the average of IBI among the groups. However, the CV_IBI_ of non-atopic patients was significantly larger than that of atopic and healthy volunteers. Patients with uncontrolled asthma had significantly higher mean LV than healthy controls. Asthmatic patients also had larger CV_LV_ compared to healthy subjects.

**Table 1:** The mean ± SD values of the average and the coefficient of variation (CV) of inter-breath interval and lung volume series.

	Inter-breath interval				Lung volume			
	Healthy	CAA	UAA	UNAA	Healthy	CAA	UAA	UNAA
**Mean**	3.51 ± 1.03	2.90 ± 0.67	3.47 ± 0.77	4.03 ± 1.15	0.64 ± 0.07	0.66 ± 0.06	0.76 ± 0.09 ^a^	0.77 ± 0.13 ^a^
**CV**	0.13 ± 0.04	0.22 ± 0.05	0.26 ± 0.06	0.51 ± 0.22 ^a^^,^ ^b^^,^ ^c^	0.27 ± 0.07	0.61 ± 0.21 ^a^	0.63 ± 0.23 ^a^	0.66 ± 0.24 ^a^

(a) p < 0.05 comparing to healthy

(b) p < 0.05 comparing to CAA

(c) p < 0.05 comparing to UAA

CAA, controlled atopic asthma; UAA, uncontrolled atopic asthma; UNAA, uncontrolled non-atopic asthma

### Non-linear analysis of IBI and LV time series

**Fig 2:**
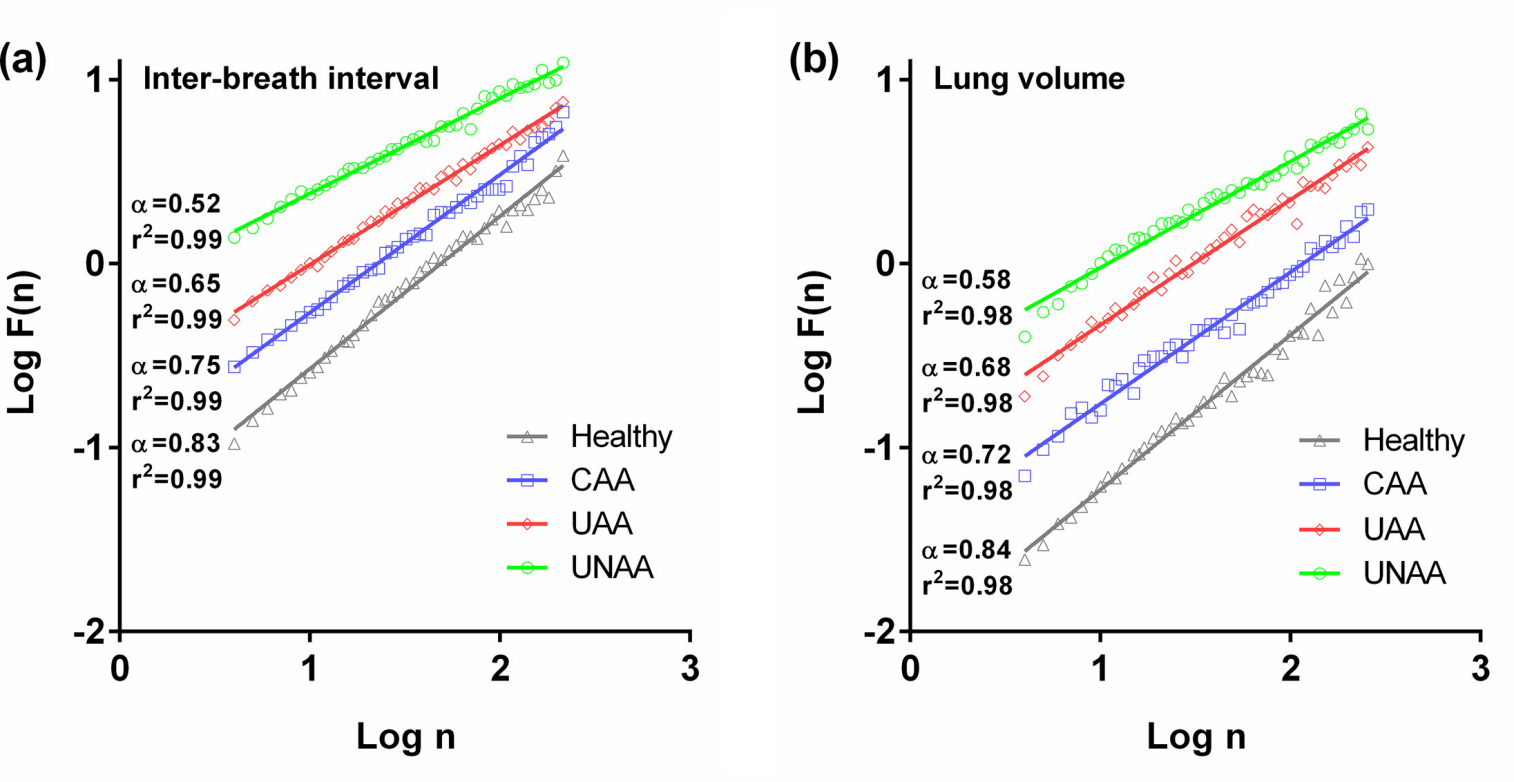
Detrended fluctuation analysis (DFA) plots for the inter-breath interval(a) and lung volume (b) time series in representative subjects. A linear relationship between log(n) and log[f(n)] indicates the presence of fractal dynamics. The scaling exponent α quantifies the strength of long-range correlations within the time series. CAA, controlled atopic asthma; UAA, uncontrolled atopic asthma; UNAA, uncontrolled non-atopic asthma.

Fig 2 shows the DFA plots for the IBI and LV time series in four representative subjects. A good linear fit can be seen (with R^2^ > 0.98) of log[F(n)] vs. log(n), which indicates the presence of fractal-like dynamics in IBI and LV time series.

**Fig 3:**
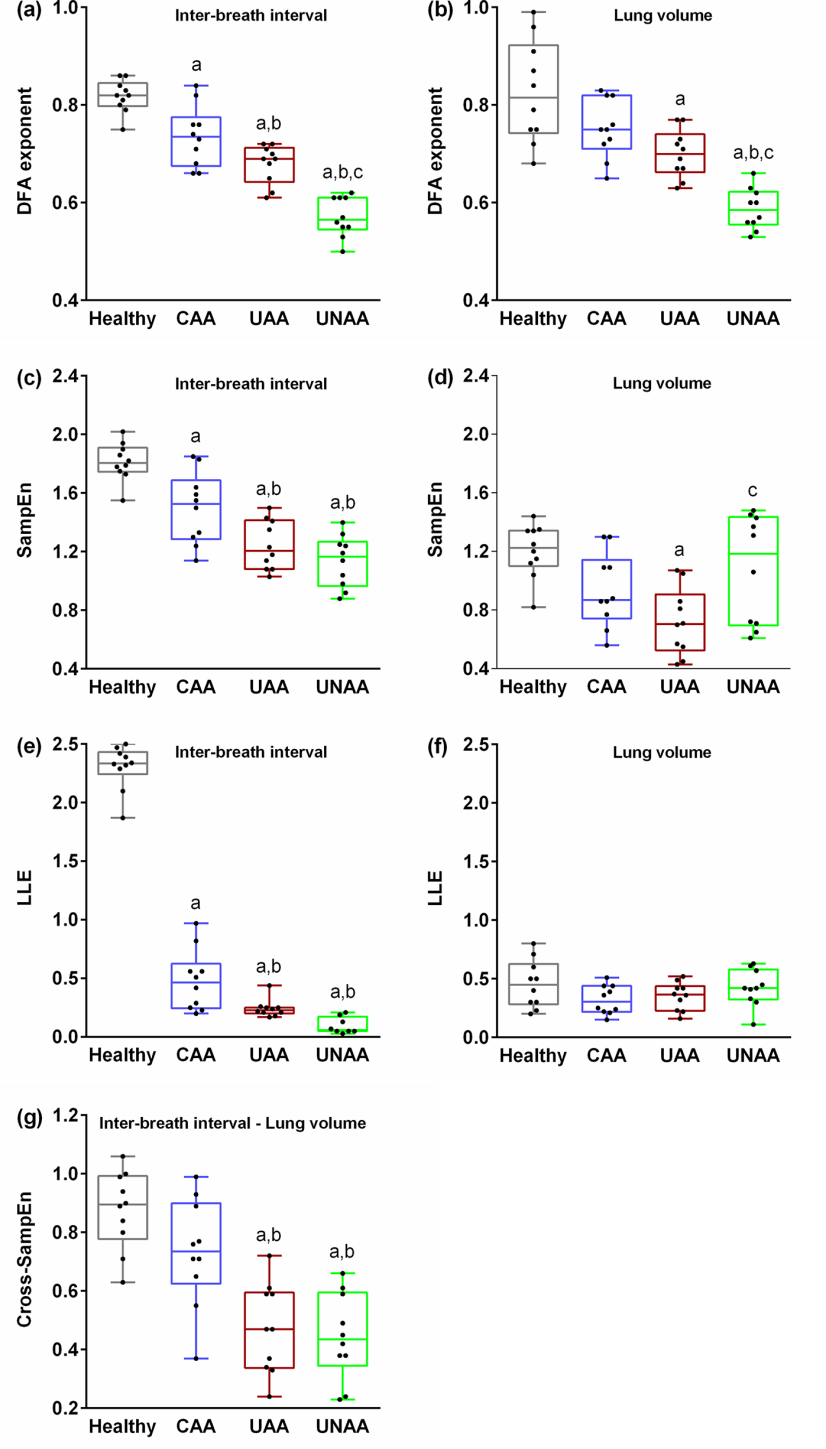
Differences of complexity indices between cases. a, p < 0.05 comparing to Healthy; b, p < 0.05 comparing to CAA; c, p < 0.05 comparing to UAA. CAA, controlled atopic asthma; UAA, uncontrolled atopic asthma; UNAA, uncontrolled non-atopic asthma; DFA, Detrended fluctuation analysis; SampEn, sample entropy; LLE, Largest Lyapunov exponents; IBI, inter-breath interval; V, volume.

The differences of complexity indices between subjects were shown in Fig 3. The α exponent was lower in the asthmatic than in the healthy subjects. Long-range correlations of both IBI (p < 0.001) and LV (p < 0.001) time-series were reduced in the asthmatic groups, especially in uncontrolled condition. Also, α values decreased significantly in the UNAA as compared with the UAA patients (IBI: p < 0.001 and LV: p = 0.004).

The SampEn_IBI_ and SampEn_LV_ decreased significantly from healthy to asthmatic subjects (p < 0.001 and p = 0.002, respectively). Unexpectedly there were no significant differences in SampEn_LV_ between the UNAA and healthy subjects (p > 0.05), whereas UNAA patients had more regular IBI series (p < 0.001). Like DFA and SampEn analysis of IBI and LV series, LLE_IBI_ was lower in the asthmatic patients (p < 0.001). However, there were no significant differences in LLE_LV_ between groups (p = 0.202). Moreover, we observed an increased synchronization between IBI and LV time-series in patients with uncontrolled asthma as appeared using cross-SampEn_IBI-LV_ analysis (p < 0.001).

### Discriminating power of nonlinear methods

**Fig 4:**
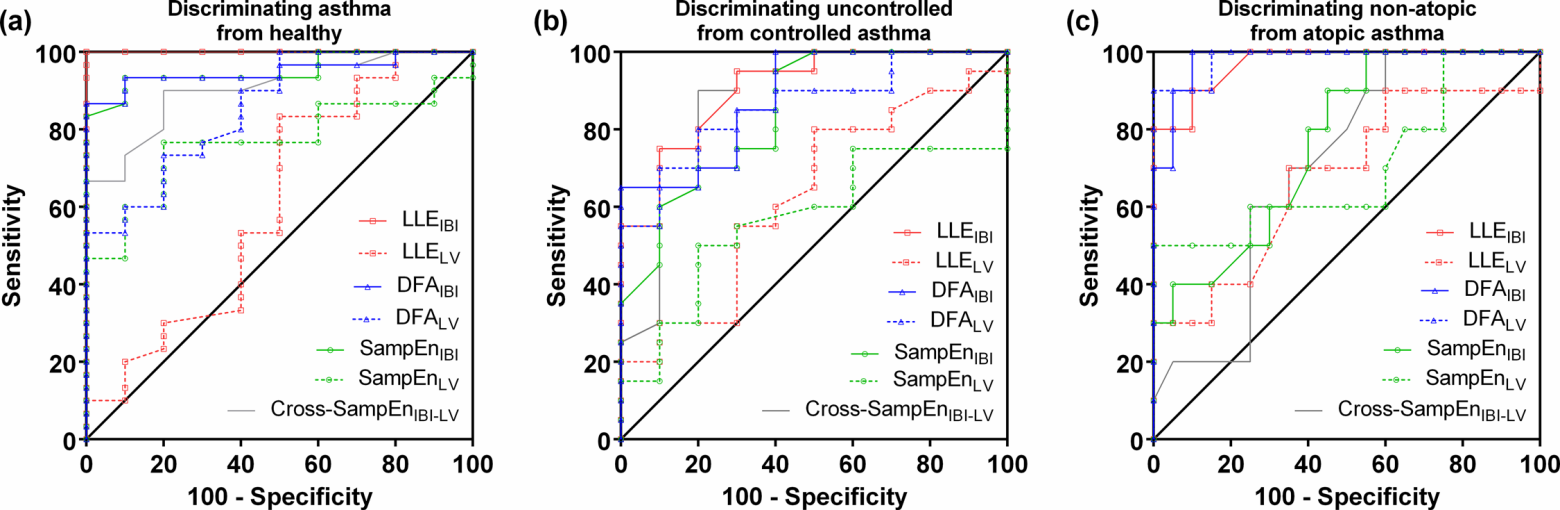
ROC curves for the ability of the complexity indices. (a), discriminating asthma from healthy; (b), discriminating uncontrolled from controlled asthma; (c), discriminating non-atopic from atopic asthma. DFA, detrended fluctuation analysis; SampEn, sample entropy; LLE, largest Lyapunov exponents; IBI, inter-breath interval; LV, lung volume.

We used ROC curve as a tool to compare the predictive value of each variability index in distinguishing the patients’ phenotype (Fig 4). The performance of the complexity indices in diagnosis of asthma based on different variability indices is described in Table 2. The LLE_IBI_ had the best discriminant ability (AUC = 1, p < 0.0001). Sensitivity and specificity of LLE_IBI_ for diagnosing asthma at a cut-off point of 1.42 was 100% (95% CI: 88–100 for sensitivity and 95% CI: 69–100 for specificity). The DFA_IBI_ (AUC = 0.95, Sensitivity = 86.7% (95% CI: 69–96), Specificity = 100% (95% CI: 69–100), p < 0.0001) and SampEn_IBI_ (AUC = 0.95, Sensitivity = 93.3% (95% CI: 78–99), Specificity = 90% (95% CI: 55–100), p < 0.0001) also had high diagnostic ability to identify asthmatic patients at a cut-off point of 0.74 and 1.69, respectively. The Cross-SampEn_IBI-LV_ (AUC = 0.90, p = 0.0002) and DFA_LV_ (AUC = 0.86, p = 0.0009) methods also gave an acceptable performance.

**Table 2:** The clinical potential of the complexity indices in discriminating asthma (n = 30) from healthy subjects (n = 10).

		AUC (95% CI)	SE	Se (95% CI) %	Sp (95% CI) %	Cut-off	p
**IBI**							
	**DFA exponent**	0.95 (0.88–1.02)	0.03	86.7 (69–96)	100 (69–100)	0.74	< 0.0001
	**SampEn**	0.95 (0.89–1.02)	0.03	93.3 (78–99)	90 (55–100)	1.69	< 0.0001
	**LLE**	1.00 (1.00–1.00)	0.00	100 (88–100)	100 (69–100)	1.42	< 0.0001
**LV**							
	**DFA exponent**	0.86 (0.73–0.98)	0.07	73.3 (54–88)	80 (44–97)	0.74	0.0009
	**SampEn**	0.77 (0.62–0.91)	0.07	76.7 (58–90)	80 (44–97)	1.11	0.0125
	**LLE**	0.61 (0.38–0.83)	0.11	83.3 (65–94)	50 (19–81)	0.50	0.3101
**Cross-SampEn (IBI-LV)**		0.90 (0.81–1.00)	0.05	90 (73–98)	80 (44–97)	0.79	0.0002

AUC, areas under the curve; SE, standard errors; Se, sensitivity; Sp, specificity; CI, confidence intervals; DFA, detrended fluctuation analysis; SampEn, sample entropy; LLE, largest Lyapunov exponents; IBI, inter-breath interval; LV, lung volume

The highest AUC (0.91) in discriminating uncontrolled from controlled asthma was obtained by using LLE_IBI_ (p = 0.0004), with 75% sensitivity (95% CI: 51–91) and 90% specificity (95% CI: 55–100) (Table 3). In this context, DFA_IBI_, Cross-SampEn_IBI-LV_, DFA_LV_ and SampEn_IBI_ also had good diagnostic ability with the AUC of 0.89 (p = 0.0007), 0.87 (p = 0.0011), 0.86 (p = 0.0015), and 0.85 (p = 0.0024), respectively. Furthermore, DFA_IBI_ had a specificity of 100% (95% CI: 69–100), but a sensitivity of 65% (95% CI: 41–85), in differentiating uncontrolled from controlled asthma at a cut-off point of 0.65.

**Table 3:** The clinical potential of the complexity indices in discriminating uncontrolled (n = 20) from controlled asthma (n = 10).

		AUC (95% CI)	SE	Se (95% CI) %	Sp (95% CI) %	Cut-off	p
**IBI**							
	**DFA exponent**	0.89 (0.76–1.01)	0.06	65 (41–85)	100 (69–100)	0.65	0.0007
	**SampEn**	0.85 (0.70–0.99)	0.08	95 (75–100)	60 (26–88)	1.47	0.0024
	**LLE**	0.91 (0.79–1.02)	0.06	75 (51–91)	90 (55–100)	0.23	0.0004
**LV**							
	**DFA exponent**	0.86 (0.73–0.99)	0.07	70 (46–88)	90 (55–100)	0.67	0.0015
	**SampEn**	0.57 (0.36–0.78)	0.11	90 (55–100)	55 (31–77)	1.33	0.5380
	**LLE**	0.63 (0.42–0.84)	0.11	80 (56–94)	50 (19–81)	0.28	0.2527
**Cross-SampEn (IBI-LV)**		0.87 (0.72–1.02)	0.08	90 (68–99)	80 (44–97)	0.63	0.0011

AUC, areas under the curve; SE, standard errors; Se, sensitivity; Sp, specificity; CI, confidence intervals; DFA, detrended fluctuation analysis; SampEn, sample entropy; LLE, largest Lyapunov exponents; IBI, inter-breath interval; LV, lung volume

The DFA_LV_ (AUC = 0.99, p < 0.0001), DFA_IBI_ (AUC = 0.98, p < 0.0001) and LLE_IBI_ (AUC = 0.97, p < 0.0001) provided high diagnostic performance in differentiating non-atopic from atopic asthma (Table 4). DFA_IBI_ (at a cut-off point of 0.63) and Cross-SampEn_IBI-LV_ (at a cut-off point of 0.69) could diagnose all 10 patients with non-atopic asthma (100% sensitivity (95% CI: 69–100)), with the specificity of 90% (95% CI: 68–99) and 40% (95% CI: 19–64), respectively. Also, the DFA_LV_ could correctly identify all 20 patients with atopic asthma (100% specificity (95% CI: 83–100) for detecting non-atopic asthma) at a cut-off point of 0.63.

**Table 4:** The clinical potential of the complexity indices in discriminating non-atopic (n = 10) from atopic asthma (n = 20).

		AUC (95% CI)	SE	Se (95% CI) %	Sp (95% CI) %	Cut-off	p
**IBI**							
	**DFA exponent**	0.98 (0.94–1.02)	0.02	100 (69–100)	90 (68–99)	0.63	< 0.0001
	**SampEn**	0.77 (0.60–0.94)	0.09	90 (55–100)	55 (31–77)	1.33	0.0186
	**LLE**	0.97 (0.92–1.02)	0.03	90 (55–100)	90 (68–99)	0.20	< 0.0001
**LV**							
	**DFA exponent**	0.99 (0.95–1.02)	0.02	90 (55–100)	100 (83–100)	0.63	< 0.0001
	**SampEn**	0.70 (0.48–0.92)	0.11	50 (19–81)	100 (83–100)	1.31	0.0749
	**LLE**	0.68 (0.46–0.89)	0.11	70 (35–93)	65 (41–85)	0.40	0.1237
**Cross-SampEn (IBI-LV)**		0.71 (0.52–0.89)	0.10	100 (69–100)	40 (19–64)	0.69	0.0713

AUC, areas under the curve; SE, standard errors; Se, sensitivity; Sp, specificity; CI, confidence intervals; DFA, detrended fluctuation analysis; SampEn, sample entropy; LLE, largest Lyapunov exponents; IBI, inter-breath interval; LV, lung volume

Table 5 demonstrates the performance of the combination of the complexity indices to the various types of asthma classification using WSRC analysis. Overall, the discriminant ability improved when indices combinations were applied; except using the combination of all indices which leads to overfitting. The combination of nonlinear analysis of IBI time-series (DFA_IBI_, SampEn_IBI_ and LLE_IBI_) appeared to have the best discriminant performance, while the corresponding ability for LV time-series is poor. Regarding the combination of IBI and LV analysis, it is also apparent that LLE performed better than DFA and SampEn in the diagnosis of various types of asthma.

**Table 5:** The clinical potential of complexity indices combination in discriminating various types of asthma.

Combination of indices*		Se (95% CI)	Sp (95% CI)	PPV (95% CI)	NPV (95% CI)	LR+ (95% CI)	LR- (95% CI)
**Discriminating asthma from healthy**							
	**DFA**_**IBI**_**, SampEn**_**IBI**_**, LLE**_**IBI**_	100 (86–100)	100 (65–100)	100 (86–100)	100 (65–100)	Inf	0 (0-NaN)
	**DFA**_**LV**_**, SampEn**_**LV**_**, LLE**_**LV**_	96.7 (81–100)	70 (35–92)	90.6 (74–97)	87.5 (47–99)	3.2 (1.3–8.3)	0.05 (0.01–0.36)
	**DFA**_**IBI**_**, DFA**_**LV**_	96.7 (81–100)	90 (54–99)	96.7 (81–100)	90 (54–99)	9.7 (1.5–62.1)	0.04 (0.01–0.26)
	**SampEn**_**IBI**_**, SampEn**_**LV**_	96.7 (81–100)	90 (54–99)	96.7 (81–100)	90 (54–99)	9.7 (1.5–62.1)	0.04 (0.01–0.26)
	**LLE**_**IBI**_**, LLE**_**LV**_	100 (86–100)	100 (65–100)	100 (86–100)	100 (65–100)	Inf	0 (0-NaN)
**Discriminating uncontrolled from controlled asthma**							
	**DFA**_**IBI**_**, SampEn**_**IBI**_**, LLE**_**IBI**_	95 (75–100)	70 (35–93)	86.4 (65–97)	87.5 (47–100)	3.2 (1.2–8.2)	0.07 (0.0–0.5)
	**DFA**_**LV**_**, SampEn**_**LV**_**, LLE**_**LV**_	95 (75–100)	60 (26–88)	82.6 (61–95)	85.7 (42–100)	2.4 (1.1–5.1)	0.08 (0.01–0.60)
	**DFA**_**IBI**_**, DFA**_**LV**_	75 (51–91)	80 (44–97)	88.2 (64–98)	61.5 (32–86)	3.8 (1.1–13.3)	0.31 (0.14–0.71)
	**SampEn**_**IBI**_**, SampEn**_**LV**_	100 (83–100)	50 (19–81)	80 (59–93)	100 (48–100)	2 (1.1–3.7)	0 (0-NaN)
	**LLE**_**IBI**_**, LLE**_**LV**_	95 (75–100)	60 (26–88)	82.6 (61–95)	85.7 (42–100)	2.4 (1.1–5.1)	0.08 (0.01–0.60)
**Discriminating non-atopic from atopic asthma**							
	**DFA**_**IBI**_**, SampEn**_**IBI**_**, LLE**_**IBI**_	90 (54–99)	100 (80–100)	100 (63–100)	95.2 (74–100)	Inf	0.10 (0.02–0.64)
	**DFA**_**LV**_**, SampEn**_**LV**_**, LLE**_**LV**_	60 (27–86)	95 (73–100)	85.7 (42–99)	82.6 (60–94)	12 (1.7–86.6)	0.42 (0.20–0.90)
	**DFA**_**IBI**_**, DFA**_**LV**_	90 (54–99)	95 (73–100)	90 (54–99)	95 (73–100)	18 (2.6–129.9)	0.11 (0.02–0.68)
	**SampEn**_**IBI**_**, SampEn**_**LV**_	70 (35–92)	95 (73–100)	87.5 (47–99)	86.4 (64–96)	14 (2–98.6)	0.32 (0.12–0.82)
	**LLE**_**IBI**_**, LLE**_**LV**_	90 (54–100)	100 (80–100)	100 (63–100)	95.2 (74–100)	Inf	0.10 (0.02–0.64)

*, weighted sparse representation based classification (WSRC) method; Se, sensitivity; Sp, specificity; PPV, positive predictive value; NPV, negative predictive value; LR+, positive likelihood ratio; LR-, negative likelihood ratio; CI, confidence intervals; Inf, infinity; NaN, not a number; DFA, detrended fluctuation analysis; SampEn, sample entropy; LLE, largest Lyapunov exponents; IBI, inter-breath interval; LV, lung volume

## Discussion

### Summary of results

We analyzed respiratory dynamics, both IBI and LV time series, using four different nonlinear methods (DFA, LLE, SampEn and Cross-SampEn) to evaluate their diagnostic capability in discriminating various types of asthma. The presented results show that all four methods have a good performance for asthma diagnosis, as well as for differentiating uncontrolled from controlled and non-atopic from atopic asthma. However, the discriminant performance improved when the combination of complexity indices of IBI were applied. The diagnostic validity of nonlinear analysis of IBI time series compared to LV time series was higher as shown by the areas under the ROC curves for each classification. The LLE_IBI_ alone as well as LLE_IBI_-LLE_LV_ and DFA_IBI_-SampEn_IBI_-LLE_IBI_ had the best discriminant ability in diagnosis of asthma with 100% sensitivity and specificity. Although the AUC of LLE_IBI_ in discriminating uncontrolled from controlled asthma was better than other methods, the combination of nonlinear analysis of IBI time series (DFA_IBI_-SampEn_IBI_-LLE_IBI_) also had a good diagnostic ability with 95% sensitivity and 70% specificity. Also, DFA_LV_ provided the best diagnostic ability in differentiating non-atopic from atopic asthma with 90% sensitivity and 100% specificity; however, DFA_IBI_ alone as well as LLE_IBI_-LLE_LV_ and DFA_IBI_-SampEn_IBI_-LLE_IBI_ presented approximately the same performance. To our knowledge, this is the first report of the classification of asthma based on the nonlinear analysis of cycle-by-cycle variations in respiration.

### Complex dynamics of respiration

Biological functions are essentially complex in nature and based on an intricate network of nonlinear dynamics and feedbacks [23]. Through these dynamical processes, biological systems perform under a delicate equilibrium which is defined by homeokinesis more precisely than previously used homeostasis [24]. Physiological parameters fluctuate continuously under non-equilibrium steady-state conditions [25] to maintain adaptability to external or internal stimuli [24]. In this context, a healthy system is stable and fluctuates normally. A shift in dynamics of a system toward either too regular or too irregular may be associated with disease state [23]. Analyzing these fluctuations carry information on the adaptability of the physiological system and may provide a new insight into the characteristics of illnesses [23]. Like all physiological systems, respiratory system is adaptive and functions in homeokinetic statuses [26]. Some states such as aging [2], hypoxia [13] and mechanical ventilation in critically ill patients [13, 27, 28, 29], shift the respiratory system toward increased regularity [23]. On the other hand, it might lose control and become unstable in some disorders like neonatal immaturity [30], panic [16, 31] and hypercapnia [10].

### Respiratory dynamics in asthma

Asthma is a common disease and has variable clinical symptoms [32]. However, many features of this disease remain largely unknown [7]. One differentiating feature of asthma from other chronic lung diseases is that it represents an episodic and complex behavior due to interaction of inflammatory, mechanical, immunological and neurological components [23, 33]. This behavior may also be associated with greater distance from steady-state equilibrium [24]. Veiga et al. demonstrated that entropy of airflow pattern is reduced in asthmatic patients and this regularity is associated with increased severity of airway obstruction [7]. A subsequent study by the same group provided evidence that respiratory impedance patterns become more regular and less complex in asthmatic patients than in healthy subjects [34]. In contrast, Gonem et al. reported an increase in the respiratory impedance entropy of asthmatic patients, which is associated with poorly controlled asthma [35]. In addition to these findings, results of our previous study showed that asthmatic patients have a significantly higher memory length in their respiratory pattern compared to healthy subjects. Memory length in this context defines the time period or scale, over which rare events within a physiological time-series do not appear randomly. This means that a rare event (e.g., tachypnea) potentially affects the respiratory rhythm of patients with asthma for longer than healthy volunteers [8].

According to previous studies, respiratory rate and breath volumes are naturally variable in cycle-by-cycle measurements [36]. Despite the growing interest in the dynamics of respiratory pattern [2, 30], few studies have investigated the nonlinear properties of IBI and LV, most probably due to technical difficulties in continuous monitoring [2, 30]. Our recording technique did not require connection to the airways, thus natural breathing was minimally disturbed. Also, the use of previously validated ANN model for the calibration of thoracoabdominal breathing movement [11], allowed us to precisely record time series of IBI and LV. Therefore, we could accurately measure the cycle-by-cycle variations in respiratory variables during prolonged and continuous respiratory monitoring. Since there has been limited information about the effect of female’s reproductive cycle on respiratory dynamics, we only used male subjects to avoid bias.

DFA analysis of IBI in our study confirmed Peng et al. [2] findings who established the fractal nature of IBI fluctuations in healthy subjects. We also found this self-similarity in LV series. Long-range correlation in a self-similar system indicates the correlation of current fluctuations’ amplitudes with potentially future values [23]. In asthmatic patients, the scaling exponents of IBI and LV shift from values close to 1 (in healthy states) toward 0.5, indicating a qualitative change in the fractal-like structure of the time/volume-series which makes it different from a physiologically relevant 1/f dynamics. We also calculated the amount of sensitivity to the initial condition in respiratory dynamics, using LLE. Largest Lyapunov exponent reflects the sensitivity to the initial conditions and the divergence of nearby points from close positions [37]. The lower LLE_IBI_ in asthmatic patients shows the more predictable characteristic of their respiratory system compared with healthy volunteers. Furthermore, we used entropy analysis to evaluate irregularity in the respiratory system. Entropy was first described by Pincus [38] as an indicator of a system’s degree of isolation; which reveals the system’s adaptability to its external environment [39]. Lower SampEn values in IBI time series of asthmatic patients show higher regularity which means less new information generation and less adaptability to its ambient universe [39]. We also performed Cross-SampEn analysis to determine the correlation of IBI and LV as two discrete but reciprocal time series [40]. Decreased Cross-SampEn in uncontrolled asthmatic patients indicates greater probability of finding similar architecture in the noticed data sets [41]. In other words, there was a strong synchronization between IBI and LV in uncontrolled asthma while respiratory time-series were more asynchronous in healthy conditions.

### Pathophysiological basis of respiratory pattern decomplexification in asthma

The physiological basis of complex dynamics of respiration is of physiological interest as well as clinical importance. Different mechanisms may be responsible for the fluctuations in respiratory periods and volumes. Although asthma is usually defined as a chronic inflammatory disease of the airways, some of its manifestations can be a result of an interaction between the immune and nervous system [42]. Previous studies showed increased levels of inflammatory mediators in different regions of the brain stem, specifically the nucleus tractus solitarius (NTS), in asthmatic subjects [42]. There is evidence of allergen-induced neuroplasticity in the NTS in animal model of asthma [43]. Chen et al. [44] demonstrated that repeated exposure to an allergen depolarizes the resting membrane potential of NTS neurons and increases spiking response to intracellular injections of depolarizing currents in allergic asthma. c-Fos activity increases as well in NTS following allergen challenge [45]. According to these alterations, which can be referred to as “central sensitization”, the intrinsic properties of brain stem neurons might change and lead to the decomplexification of respiratory pattern in asthma [42, 44]. Apart from NTS, suprapontine and chemoreceptor factors might be involved in modulation of the respiratory dynamics in patients with asthma [39]. Previous studies reported some alterations in respiratory mechanics such as airway narrowing and increased airway resistance in a periodic and widespread manner in asthma [46]. Hence, reduced complexity in the respiratory pattern in asthma may be partly because of the lower degree of freedom of the respiratory system. We did not study central or peripheral neural activity in our study and these issues can be looked at in future investigations.

### Controlled vs. uncontrolled asthma

Lung function test is the most objective measure of asthma severity and its response to therapy [33]. Although single-point assessments or mean values are regularly used to quantify lung function, they lack plenty of information that can be extracted from monitoring fluctuations in lung function over time [6, 3]. Therefore, measures of variability amplitude have been recommended as an additional aspect of asthma control assessment [47]. Recent studies established that increased PEF (Peak Expiratory Flow) variability over long term may be suggestive of worsening and poorly controlled asthma [3]. Variability analysis over time has been applied to other features of respiratory function, such as oscillatory resistance. Que and colleagues reported that calculation of respiratory system impedance using the FOT (Forced Oscillation Technique) may be able to predict following airway narrowing over the next 24 hours [24]. Gonem and colleagues have also found that increased impedance, heterogeneity of impedance, and fluctuation of heterogeneity of impedance over time were associated with poorly controlled asthma [35]. Temporal fluctuations in lung function may perhaps be self-similar at multiple time scales [23]. Thus, relatively short term fluctuations can be used to provide insights into lung function variability over longer time scales [35] for consequently indicating asthma control [33]. Indeed, an eminent example is the IBI that exhibit distinctive scaling exponents [48]. Our previous study resulted in significantly higher memory length of IBI series in uncontrolled asthmatic patients compared to healthy subjects. This indicates longer effects of rare events (e.g. tachypnea) in the respiratory rhythm of patients with uncontrolled asthma [8]. The presented results also show a strong synchronization between IBI and LV series and a reducing in the long range correlation, irregularity and chaotic nature of respiratory dynamic in uncontrolled asthma. Moreover, we could differentiate uncontrolled from controlled asthma with 100% sensitivity (95% CI: 80–100) and 90% specificity (95% CI: 54–99) using the combination of nonlinear analysis of IBI time series (DFA_IBI_-SampEn_IBI_-LLE_IBI_), as the best performance. It seems that analyzing short term cycle-by-cycle variations in respiration can be useful in clinic in order to predict asthma control and alter therapy accordingly to prevent exacerbations and maintain clinical stability.

### Atopic vs. non-atopic asthma

Asthma is traditionally divided into two main categories: atopic and non-atopic [49]. These phenotypes have different pathophysiology in terms of relationships between airway inflammation, lung function, and bronchial hyper-responsiveness [50]. Physicians distinguish atopic from non-atopic asthma based on the results of skin tests and clinical symptoms. Atopic asthma is an IgE mediated allergic reaction, which is accompanied by infiltration of eosinophils in the lung [51]. Inhibition of airway eosinophil survival with inhaled glucocorticoids is also its basis of therapy [52]. Non-atopic asthma has been first described as ‘intrinsic asthma’ with later onset in life, higher degree of severity and female predominance [53]. It is characterized by persistent airway neutrophilia [54] in which eosinophilic inflammation is almost absente [55]. This is why several studies revealed poor responsiveness to inhaled glucocorticoids [54]—although there are conflicting data in this area [56, 57]. Also, depression or anxiety symptoms in children are associated with non-atopic asthma [58] but not with atopic asthma [58]. In consistent with clinical observations, we found significant differences in respiratory dynamics between non-atopic and atopic asthma, possibly reflecting different pathophysiological mechanisms. Patients with non-atopic asthma exhibited reduced respiratory pattern complexity, including decreased long range correlation, irregularity and sensitivity to initial conditions, compared to patients with atopic asthma. This significant lower complexity is justified by a higher degree of disease severity in non-atopic asthma [53]. Our results showed high diagnostic performance of complexity indices in differentiating non-atopic from atopic asthma. DFA_LV_ provided the best diagnostic ability with 90% sensitivity and 100% specificity at a cut-off point of 0.63. This is, to our knowledge, the first report of distinguishing between atopic and non-atopic asthma just based on respiratory dynamics.

## Conclusions and perspectives

We measured the cycle-by-cycle variations of IBI and LV variables and found that these fluctuations show decreased long range correlation, irregularity and sensitivity to initial conditions in asthmatic patients, particularly in uncontrolled state. Nonlinear analyses of respiratory dynamics have been indeed shown useful in asthma diagnosis, as well as in differentiating uncontrolled from controlled and non-atopic from atopic asthma. The presented results might shed new light not only to understand different pathophysiological mechanisms of asthma, but may also provide diagnostic and prognostic information to make personalized predictions in guiding therapy. For instance, despite the fact that clinicians distinguish controlled from uncontrolled asthma over time, nonlinear analyses of respiratory dynamics make it possible to differentiate them much earlier. Furthermore, analyzing respiratory dynamics may provide information about the patients’ response to treatment and may be used in clinical practice as well as for home-based monitoring of disease progression. However, future studies are needed to be performed on females as well as males in multiple centers with more subjects to increase the validity of the study and to improve these novel methods for better patient management.

## Supporting Information

S1 File Classification method. (DOCX)
