# Supplementary material for: Classification of Asthma Based on Nonlinear Analysis of Breathing Pattern
Source: PLoS One. 2016 Jan 29;11(1):e0147976. doi: 10.1371/journal.pone.0147976 (PMC4732950; doi:10.1371/journal.pone.0147976)
Supplement: S1 File — (DOCX) [file pone.0147976.s001.docx]

**Classification method**

**1. Sparse Representation based-Classification (SRC)**

There currently exist many conventional classification methods such as SVM, KNN, NS and etc. Sparse Representation based-Classification (SRC) was first used in face recognition [1]. This method is similar to Nearest Subspace method (NS) [1]. Therefore, SRC classifier does not need any preprocessing of the training sample set prior to their use [1]. SRC classification approach assigns sample vector $y$ as an input*,* which belongs to an unknown class. This idea is extended to SRC when vector $y$ is being assigned to the class that is represented by training samples and is related to coefficients of sparse representation of $y$ in the best way [1]. Suppose:

$$y=Ax (1)$$

where $A$ is a $m \times n$ matrix it describes training sample with vectors $a_{k}$ as columns; $A$ includes features as rows and samples as columns. Also, $x$ is a $n\times1$ coefficients vector and it needs to be sparse. The favorite situation corresponds to a difficult underdetermined problem in which $n$ (the number of test samples) is much more than $m$ (the number of features). This problem is NP-hard and has no accurate answer practically [2]. Therefore, to get the sparsest solution for (1)**,** the solution needs to have the minimal $\mathcal{l}_{0}$-norm. This search to find the minimal $\mathcal{l}_{0}$-norm is an intractable problem. Therefore, researchers look for the tractable method to solve (1). Some algorithm were suggested like $\mathcal{l}_{1}$-norm minimization [3] and matching pursuit [4]. In this paper, Smoothed $\mathcal{l}_{0}$-norm (SL0) methods is used to solve $(1)$ [5]. The cost function of SL0 defines as:

$$\left\| x \right\|_{0}\approx n-F_{\sigma}\left( x \right)=n-\sum_{k=1}^{n} e^{-\frac{x_{k}^{2}}{2\sigma^{2}}} (2)$$

In this method, $\sigma$ approaches zero iteratively. Hence, below problem must be solved to find the sparsest solution for (1):

$$\hat{x}=\arg\min_{x} \left\| x \right\|_{0}\approx\arg\max_{x} F_{\sigma}\left( x \right) subject to y=Ax (3)$$

**1.1 Decision methods**

Since practical experiments have some errors due to noise and modeling error, the classifier cannot represent test samples well [1]. For decision making, many methods were suggested that use the training samples collection. In this paper ‘Subspace method’ is chosen [1].

The method for decision making finds the best representation for $y$ which reproduces test sample by related group (training) samples linearly in the most proper way [1]. Therefore class label of $y$ will be found in below equation:

$${i^{*}=\arg\min_{i\in\left\{ 1,2,\ldots,c \right\}} \left\| y-A\delta_{i}\left( \hat{x} \right) \right\|}_{2}^{2} (4)$$

In this case $\delta_{i}\left( . \right)$ is the characteristic function and $\delta_{i}\left( \hat{x} \right)=[0,\ldots,0,\hat{x}_{i,1},\hat{x}_{i,2},\ldots,\hat{x}_{i,n_{i}},0,\ldots,0]$. $n_{i}$ is the number of training samples in $i^{th}$ class.

**2. Weighted sparse representation based-classification (WSRC)**

Discrimination capability of SRC is lost in datasets which distribute in the same direction [6]. Data on same direction distribution means that the samples with the same vector directions are members of different classes [6]. SRC requires normalizing the samples and leads to map the samples onto hypersphere [6]. Therefore, data with the same direction distribution are not separable. Although the mentioned normalization is ineffective on the solution of SRC performance, it is an inseparable section of SRC algorithm. Weighted sparse representation based-classification (WSRC) remedies the drawback of SRC and its performance improves by adding the weights [7]. We suggested using the Minkowski distance between the new sample $y$ and the related training samples as weight. We considered $M=\infty$ for Minkowski distance (It also calls $maximum norm$ distance) and weights will be calculated as:

$$w_{k}=\max\left| y-a_{k} \right| (5)$$

where $a_{k}$ is the $k^{th}$ training sample. Therefore, weight vector $w$ is defined as $w=\left[ w_{1},w_{2},\ldots,w_{n} \right]^{T}$. Hence, the optimization problem (3) can be written as:

$$\hat{x}=\arg\min_{x} {\sum_{k=1}^{n} w_{k}\left| x_{k} \right|}_{0} subject to y=Ax (6)$$

The solution process of new optimization problem changes in reconstruction technique so that SL0 should be replaced with ‘Weighted SL0’. Cost function of weighted SL0 is defined as:

$${\sum_{k=1}^{n} w_{k}\left| x_{k} \right|}_{0}\approx\sum_{k=1}^{n} w_{k}-\sum_{k=1}^{n} w_{k}e^{-\frac{x_{k}^{2}}{2\sigma^{2}}} (7)$$

**3. References**

1. Wright J, Yang AY, Ganesh A, Sastry SS, Ma Y. Robust face recognition via sparse representation. IEEE Trans Pattern Anal Mach Intell. 2009 Feb;31(2):210-27. doi: 10.1109/TPAMI.2008.79.
2. Donoho DL. Compressed sensing. IEEE Trans Inf Theory. 2006 Apr;52(4):1289-1306. doi: 10.1109/TIT.2006.871582
3. Donoho DL. For most large underdetermined systems of linear equations the minimal 1-norm solution is also the sparsest solution. Commun Pure Appl Math. 2006 Jun;59(6):797-829.
4. Krstulović S, Gribonval R. Mptk: Matching pursuit made tractable. in Proc. ICASSP 2006 (3):496–499.
5. Oxvig CS, Pedersen PS, Arildsen T, Larsen T. Improving smoothed l0 norm in compressive sensing using adaptive parameter selection. 2012 arXiv preprint arXiv:1210.4277.
6. Yin J, Liu Z, Jin Z, Yang W. Kernel sparse representation based classification. Neurocomputing. 2012 Feb 1;77:120-128.
7. CY Lu, H Min, J Gui, L Zhu, YK Lei. Face recognition via weighted sparse representation. J Vis Commun Image Represent. 2013 24(2):111-116.
